# Dysphagia following prolonged mechanical ventilation and tracheostomy in critical ill patients. results of edisval study pilot phase

**DOI:** 10.1186/2197-425X-3-S1-A677

**Published:** 2015-10-01

**Authors:** A Fernández Carmona, I Macías Guarasa, R Gutiérrez Rodríguez, P Martínez López, MA Díaz Castellanos, EDISVAL Group

**Affiliations:** Virgen de las Nieves Hospital, Granada, Spain; Hospital Carlos Haya, Malaga, Spain; Hospital Virgen de la Victoria, Malaga, Spain; Hospital Santa Ana, Motril, Spain

## Introduction

Available data shows that dysphagia and swallowing disorder rate secondary to artificial airway and prolonged mechanical ventilation in critical tracheostomized patients is high (50-83%), nevertheless it real incidence is not yet well established. Dysphagia is directly related to bronchial aspirations and respiratory infections. The rate of respiratory infections on tracheostomized patients is also very high (Some series next 100%). The re-establishment of airway using speaking valves allow the rehabilitation and post-recovery of those disorders, as well as deglutition and phonatory system rehabilitation.

The aim of EDISVAL Study is to determine the usefulness of speaking valve in preventing respiratory nosocomial infections in critical tracheostomized patients diagnosed of dysphagia secondary to artificial airway, for what it was done a screening test of dysphagia to the critical tracheostomized patients.

## Objectives

To describe the incidence of swallowing disorder secondary to artificial airway and prolonged mechanical ventilation in critical patients who require tracheostomy.

## Methods

From September 2014 until December 2014, in all patients over 18 years, during mechanical ventilation weaning-decannulation phase, without neurological or surgical disease which could contribute to the appearance of dysphagia, there was realized the Modified Evans Blue Dye Test (MBDT) as dysphagia screening test. This study was carried out of simultaneous form in 7 intensive care units including first, second and third level centers.

## Results

Mean age average of the patients was 71,6 years, the initial APACHE´S average was 21.2. Mean time of mechanical ventilation was 23,6 days. During the study period we studied 29 patients by MEBDT, 27 patients were diagnosed of dysphagia, and included to the EDISVAL study; we did not carry out other specifics test of dysphagia to discriminate a possible MEBDT false negative.

In the patients with negative MEBDT, not diagnosed of dysphagia, respiratory infections were not registered.

In the patients diagnosed of disfagia included at EDISVAL study, in spite of strict measures for it prevention, including absolute oral diet, 7 respiratory infections were registered, 2 catalogued as tracheobronchitis and 5 as pneumonia. 3 of the patients who suffered respiratory serious infections died during hospital stay.

## Conclusions

The dysphagia secondary to artificial airway incidence in our series was 93,4% according to the Modified Evans Blue Dye Test. Respiratory infections were a frequent complication in the patients diagnosed of dysphagia, with a probable repercussion in the mortality of the patients.
